# Validating a functional near-infrared spectroscopy diagnostic paradigm for Major Depressive Disorder

**DOI:** 10.1038/s41598-020-66784-2

**Published:** 2020-06-16

**Authors:** Syeda Fabeha Husain, Rongjun Yu, Tong-Boon Tang, Wilson W. Tam, Bach Tran, Travis T. Quek, Shi-Hui Hwang, Cheryl W. Chang, Cyrus S. Ho, Roger C. Ho

**Affiliations:** 1 Institute for Health Innovation and Technology (iHealthtech), National University of Singapore, Singapore, 117599 Singapore; 2 Department of Psychological Medicine, Yong Loo Lin School of Medicine, National University of Singapore, Singapore, 119228 Singapore; 3 Department of Psychology, Faculty of Arts and Social Science, National University of Singapore, Singapore, 119077 Singapore; 4 Centre for Intelligent Signal and Imaging Research (CISIR), University Teknologi PETRONAS, 32610 Seri Iskandar, Perak Darul Ridzuan, Malaysia; 5 Alice Lee Centre for Nursing Studies, Yong Loo Lin School of Medicine, National University of Singapore, Singapore, 117597 Singapore; 6 Johns Hopkins Bloomberg School of Public Health, Johns Hopkins University, Baltimore, MD 21205 USA; 7 Institute for Preventive Medicine and Public Health, Hanoi Medical University, Hanoi, Vietnam; 8 Center of Excellence in Behavioral Medicine, Nguyen Tat Thanh University, Ho Chi Minh City, Vietnam; 9 Department of Psychological Medicine, National University Health System, Singapore, 119228 Singapore

**Keywords:** Neuroscience, Biomarkers

## Abstract

Reduced haemodynamic response in the frontotemporal cortices of patients with major depressive disorder (MDD) has been demonstrated using functional near-infrared spectroscopy (fNIRS). Most notably, changes in cortical oxy-haemoglobin during a Japanese phonetic fluency task can differentiate psychiatric patients from healthy controls (HC). However, this paradigm has not been validated in the English language. Therefore, the present work aimed to distinguish patients with MDD from HCs, using haemodynamic response measured during an English letter fluency task. One hundred and five HCs and 105 patients with MDD took part in this study. NIRS signals during the verbal fluency task (VFT) was acquired using a 52-channel system, and changes in oxy-haemoglobin in the frontal and temporal regions were quantified. Depression severity, psychosocial functioning, pharmacotherapy and psychiatric history were noted. Patients with MDD had smaller changes in oxy-haemoglobin in the frontal and temporal cortices than HCs. In both regions of interest, oxy-haemoglobin was not associated with any of the clinical variables studied. 75.2% and 76.5% of patients with MDD were correctly classified using frontal and temporal region oxy-haemoglobin, respectively. Haemodynamic response measured by fNIRS during an English letter fluency task is a promising biomarker for MDD.

## Introduction

Major depressive disorder (MDD) is a common global disorder that impairs daily living for sufferers, and causes significant societal and economic burden^1,2^. It is characterised by low mood and anhedonia for almost every day in a two-week period. These core symptoms are often accompanied by several other somatic, psychological and cognitive symptoms, that vary between individuals in terms of their occurrence and severity^3^. Patients are assessed during clinical interviews, but given the heterogenous nature of this disorder, subjective patient reports, and reliance on individual clinical judgement, there is poor inter-rater reliability in diagnosing MDD^4^. Despite the disease burden, and inaccuracies of clinical interviews, there are currently no quantitative biological tests for MDD. There is however, growing evidence for the utility of functional neuroimaging, particularly functional near-infrared spectroscopy (fNIRS), to aid in the diagnosis of MDD.

Light in the NIR spectrum has the unique property of penetrating tissues, and being preferentially absorbed by haemoglobin^5^. The absorbance spectra of haemoglobin is dependent on its binding with oxygen, which enables fNIRS devices to continuously monitor both oxy-haemoglobin and deoxy-haemoglobin in the cerebral cortex^6^. NIRS signals are a surrogate measure of the underlying neural activity, described by a phenomenon known as neurovascular coupling^7^. Regional neuron activity triggers an increase in blood flow and volume that is disproportionately higher than the metabolic demand. Therefore, cerebral haemodynamic response typically involves a nett increase in oxy-haemoglobin, and a simultaneous slight decrease in deoxy-haemoglobin^8^. Since the changes in oxy-haemoglobin are greater than deoxy-haemoglobin, the former is used as an indicator of cerebral activity^9^. Although NIR light cannot reach subcortical regions, the practical advantages of fNIRS make it a suitable tool for assessing psychiatric patients. Compared to conventional functional neuroimaging methods, such as functional magnetic resonance imaging (MRI) and positron emission tomography (PET), fNIRS is cost-effective and does not involve ionising radiation, restraints or loud noise^10^.

fNIRS studies have consistently reported reduced oxy-haemoglobin in the frontotemporal cortices during the verbal fluency test (VFT) in patients with MDD compared to healthy controls (HC)^11^. This cognitive task assesses language production and word retrieval controlled by the posterior inferior frontal gyrus, and semantic processing controlled by the anterior inferior temporal gyrus^12^. Thus, from a neuropsychological perspective, reduced frontotemporal haemodynamic response to cognitive stimuli may be associated with the cognitive symptoms of MDD. At the same time, reduced haemodynamic response may reflect neurophysiological changes associated with MDD. Although the biological mechanism of haemodynamic dysfunction is not clear, post-mortem and animal models suggest neurons and glial cells responsible for maintaining haemodynamic response lose their function in patients with MDD^13^.

While several variations of the VFT have been published in the fNIRS literature^14–17^, the VFT and NIRS signal processing protocol proposed by Takizawa *et al*.^18^ was designed specifically for neuropsychiatric assessment. Subsequent studies using this protocol have demonstrated that frontotemporal oxy-haemoglobin response magnitude could distinguish HCs and patients with common psychiatric disorders, namely MDD, schizophrenia and bipolar disorder^19^. Moreover, frontotemporal oxy-haemoglobin response magnitude is associated with depression severity^20^, social functioning^21^ and family psychiatric history^22^ in patients with common psychiatric disorders. Despite these promising results, this paradigm has not been modified for the English language. Hence, the objective of this study is to validate an fNIRS diagnostic test for MDD in English-speaking adults.

We hypothesise that low activation in the frontotemporal cortex is a salient trait of patients with MDD, and that frontotemporal oxy-haemoglobin response magnitude can differentiate patients with MDD from HCs with acceptable accuracy. Hence, the first aim of this study was to demonstrate lower frontotemporal oxy-haemoglobin response magnitude during an English version of the VFT in patients with MDD compared to HCs. The second aim was to investigate the relationship between cortical oxy-haemoglobin and several clinical factors such as depression severity, pharmacotherapy and clinical history. Lastly, we report the sensitivity and specificity of classifying patients with MDD and HCs with frontotemporal oxy-haemoglobin response magnitude.

## Results

### Demographic and clinical data

HCs and patients with MDD did not differ in age, gender, ethnicity, handedness and family psychiatric history (p > 0.05) (Table 1). Compared to controls, patients with MDD had fewer years of education [*t* = 5.28, *df* = 186.46, *p* ≤ 0.001, *d* = 0.73, 95% CI, (1.08 to 2.37)], generated fewer words [*t* = 4.2, *df* = 203.62, *p* ≤ 0.001, *d* = 0.63, 95% CI, (1.89 to 5.24)], had higher HAM-D scores [*t* = -15.9, *df* = 123.33, *p* ≤ 0.001, *d* = 2.19, 95% CI, (-13.79 to -10.73)] and lower GAF scores [*t* = 17, *df* = 176.21, *p* ≤ 0.001, *d* = 2.35, 95% CI, (21.72 to 27.43)]. Approximately 70% of patients had no prior admission to a psychiatric ward, and approximately 35% were medication naïve. Amongst patients with MDD receiving pharmacotherapy, majority were on antidepressants, and a fraction were on combination anxiolytics or antipsychotics (Supplementary Table 2).

**Table 1:** Demographic and clinical characteristics.

	MDD (n = 105)	HC (n = 105)	*p*-value
Age (years)	36.2 ± 13	36.4 ± 13	0.915
Gender			0.574
Male	45 (42.9%)	40 (38.1%)	
Female	60 (57.1%)	65 (61.9%)	
Ethnicity			0.052
Chinese	86 (81.9%)	80 (76.2%)	
Malay	10 (9.5%)	5 (4.8%)	
Indian	7 (6.7%)	8 (7.6%)	
Eurasian	0	3 (2.9%)	
Others	2 (1.9%)	9 (8.6%)	
Handedness †			0.235
Right	66 (88%)	89 (90.8%)	
Left	8 (10.7%)	5 (5.1%)	
Ambidextrous	1 (1.3%)	4 (4.1%)	
Education (years)	14.8 ± 2.7	16.5 ± 1.9	**≤0.001**
Number of words generated	16.6 ± 6.6	20.2 ± 5.7	**≤0.001**
HAM-D	14.7 ± 7.6	2.4 ± 2.3	**≤0.001**
GAF	68.9 ± 12.5	93.4 ± 7.9	**≤0.001**
Family psychiatric history †	27 (26.5%)	17 (18.3%)	0.230
Age at onset (years)	30.8 ± 11.4	—	
Duration of illness (years)	5.3 ± 6.5	—	
Past admission to psychiatric ward	32 (30.5%)	—	
Pharmacotherapy	68 (64.8%)	—	
Fluoxetine equivalent dose (mg/day)	28.9 ± 17.6	—	
Diazepam equivalent dose (mg/day)	6.2 ± 5	—	
Chlorpromazine equivalent dose (mg/day)	192.9 ± 132.6	—	

Mean ± SD are shown and *p*-values ≤0.05 are in bold.

^†^Complete demographic data was not obtained for all subjects (Known handedness in healthy controls, n = 98; in major depressive disorder, n = 75. Known family history of psychiatric illness in healthy controls, n = 93; in major depressive disorder, n = 102).

### Comparing haemodynamic response between controls and patients

**Figure 1:**
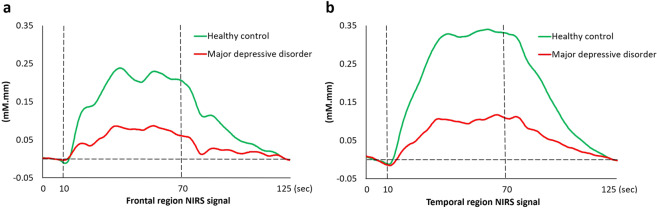
Average oxyhaemoglobin waveforms in the (**a**) frontal and (**b**) temporal regions.

HCs and patients with MDD did not differ in the number of available channels in the frontal (MDD, n = 101; HC, n = 101; *p* = 0.094) and temporal (MDD, n = 102; HC, n = 103; *p* = 0.952) regions. Patients had lower integral values than controls in both the frontal [*t* = 4.76, *df* = 200, *p* ≤ 0.001, *d* = 0.67, 95% CI, (39.24 to 94.81)] and temporal [*t* = 6.99, *df* = 199.42, *p* ≤ 0.001, *d* = 0.98, 95% CI, (68.16 to 139.65)] regions (Fig. 1), but centroid values did not differ between controls and patients (frontal region, *p* = 0.567; temporal region, *p* = 0.689).

### Factors associated with haemodynamic response

The frontal region integral value was not associated with demographic factors, clinical factors and channel availability for both HCs and patients with MDD. Similarly, the temporal region integral value was not associated with demographic and clinical factors for HCs and patients with MDD (supplementary table 3 and 4). However, the temporal region integral value negatively correlated with the number of available channels amongst HCs (*r* = −0.222, *p* = 0.024), but not patients with MDD. Stepwise linear regression also revealed associations between temporal region integral value and number of available channels amongst HCs (β = −6.68, SE = 2.92, *p* = 0.024, adjusted R^2^ = 0.04). Despite this statistically significant association, the number of available channels only accounts for 4% variability in temporal integral value. Thus, both the frontal and temporal integral values were used in ROC analysis.

### Differentiating controls and patients with major depression

**Figure 2:**
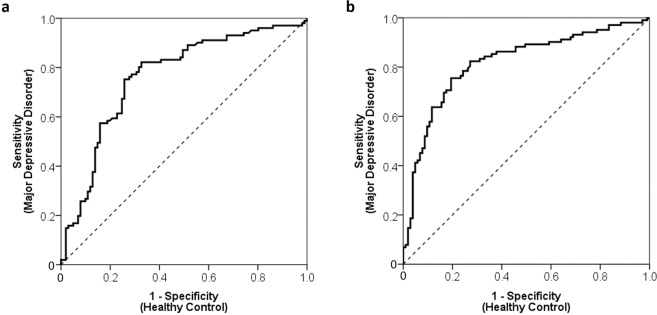
Receiver operating characteristic analysis of the (**a**) frontal and (**b**) temporal region integral values between patients with MDD and HCs.

The area under the ROC curve for the frontal and temporal region integral values, were 0.76 [95% CI, (0.7 to 0.83)] and 0.82 [95% CI, (0.76 to 0.88)] respectively (Fig. 2). Using an optimal threshold value of 51, the frontal region integral value correctly classified 75.2% of patients with MDD (proportion of patients/measurements: 76/101) and 74.3% of HCs [proportion of controls/measurements: 75/101; positive predictive value (PPV) = 0.75; negative predictive value (NPV) = 0.75]. Similarly, with an optimal threshold value of 81, the temporal region integral value correctly classified 76.5% of patients with MDD (78/102) and 76.7% of HCs (79/103; PPV = 0.77; NPV = 0.77; Table 2).

**Table 2:** Sensitivities and specificities of frontal and temporal region integral values.

Integral value	Frontal region		Temporal region	
	Sensitivity	Specificity	Sensitivity	Specificity
120	0.91	0.34	0.88	0.54
110	0.91	0.42	0.86	0.57
100	0.90	0.44	0.85	0.64
90	0.87	0.51	0.83	0.68
80	0.83	0.55	0.76	0.78
70	0.82	0.60	0.70	0.83
60	0.79	0.68	0.66	0.84
50	0.74	0.74	0.64	0.86
40	0.65	0.75	0.56	0.90
30	0.57	0.83	0.48	0.91
20	0.48	0.86	0.41	0.95
10	0.38	0.87	0.34	0.96

## Discussion

Results from this study lend support for an fNIRS paradigm as a supplementary tool for MDD diagnosis. In addition to patients with MDD having significantly lower integral values than HCs, integral values were not associated with any of the demographic and clinical factors. In particular, the independence of integral values from illness duration and treatment with psychotropic medications, suggests that the proposed fNIRS paradigm may be used to assess patients at any stage of their disorder. A similar conclusion was made in a recent publication on patients at various stages of psychosis^23^. Additionally, the independence of integral values from the number of words generated during the VFT and years of education, suggests that the proposed test is suitable for most people with and without MDD.

Another significant finding in this study is that the integral values could distinguish HCs and patients with MDD with acceptable sensitivity and specificity. The classification rates reported in this study are similar to an earlier report on Japanese subjects^19^. Our finding provides a cross-cultural validation of fNIRS metrices, as a marker for common disorders like MDD^24^. However, the optimal cut-off integral values in our sample were lower than the Japanese study^19^. This variation may be due to differences in the design of the VFT. While we used English alphabets, the Japanese VFT utilises syllabary. Since lexical retrieval strategies differ between alphabetic and non-alphabetic languages^25^, it may have an effect on cortical activity. Likewise, amongst fNIRS studies done on Chinese-speaking patients with MDD, cortical activity in the temporal region differs between HCs and patients during the Chinese phonological VFT^26^, whereas differences in prefrontal cortex activity was observed during the Chinese categorical VFT^27^. Furthermore, variations of the Chinese letter fluency task have been proposed in fNIRS studies on patients with schizophrenia, because different populations use different syllable systems^28^. Specifically, the letter fluency task designed by Quan *et al*.^29^ for subjects in mainland China differed from that used by Chou *et al*.^30^ for subjects in Taiwan. Hence, language specific validation of this fNIRS paradigm is important.

In research and clinical settings alike, the presence and severity of depressive symptoms are typically assessed by instruments like the HAM-D and GAF. Although we did not observe an association between these scales and integral values, this association was previously reported. However, the methodological differences between studies, and limitations of these scales should be noted. For instance, Kawano *et al*.^20^ recruited patients with various psychiatric disorders, and found that frontal region integral values negatively correlated with the total score of the 21-item HAM-D. The HAM-D can differentiate symptomatic and remitted patients with excellent accuracy^31^, but its discriminatory power for mild, moderate and severe depression is lower^32^. Moreover, the recommended threshold values for depression severity categories vary between publications^33^. Hence, total HAM-D scores may not be a reliable measure of depression severity for a correlation analysis with integral values. Still, negative correlation between oxy-haemoglobin and individual HAM-D items have been reported, albeit in differ cortical region of interest. Items include depressed mood, work and activities^34^, insomnia early, psychomotor retardation^35^, and suicidal ideation^36^. Yet, the options for many HAM-D items do not vary as a function of depression severity. Thus, individual item scores may not be a reliable indicator for disorder severity^37^. The level of oxy-haemoglobin measured at individual channels of patients with MDD has also been linked to the GAF^38^. The GAF score is a single measurement of overall impairment, that takes psychological symptoms and social and occupational functioning into consideration. However, much like the HAM-D items, the GAF scale also lacks continuity between intervals^39^. Nevertheless, low frontotemporal cortical activity is associated with the syndromic criteria for MDD in the fifth edition of the Diagnostic and Statistical Manual of Mental Disorders (DSM-5)^11^.

Low frontal and temporal region integral values may represent a decline in physiological function amongst MDD patients. Concerted evidence from other neuroimaging modalities and post-mortem studies indicate that pathophysiological changes occur in the prefrontal cortex. Compared to HCs, neuron size and glia cell density are reduced in patients with MDD^40^. MRI techniques reveal grey matter volume reduction^41^ and blood-oxygen-level-dependent response attenuation during the VFT^42^. Furthermore, a previous study using PET reported reduced blood flow and metabolism in the prefrontal cortex of patients with MDD^43^. Biomarker validation and pathophysiology elucidation for MDD is especially challenging, since there are no established biological gold standards. Regrettably, both psychological and somatic symptoms can only be evaluated by clinical interviews. Therefore, psychiatric nosology may dampen the classification accuracy of potential biomarkers^44^. On the other hand, low integral values may be particularly useful to evaluate the cortical function of psychiatric patients who may underreport their symptoms, remitted patients at risk of relapse^45,46^ and patients with poor prognosis^26,47–49^.

This study has several limitations. Firstly, this cross-sectional study could not establish a causal relationship between low frontotemporal activity and onset of MDD. In contrast, previous longitudinal studies reported concurrent oxy-haemoglobin increase and MDD severity decrease^50,51^. However, these changes were only seen in 1 or 2 channels located at the temporal cortex. Therefore, integral value for the frontal and temporal regions may not vary over time. Longitudinal studies focussing on the same regions of interest are needed to verify this hypothesis. Moreover, consistency of frontal and temporal region integral values at different time points would demonstrate internal validity. Secondly, MDD subtypes were not compared in this study, even though it is a heterogenous disorder^52^. This may explain why clinical outcomes were not associated with integral values in this study. Future fNIRS investigations on other clinical outcomes, such as treatment response, may identify differences between MDD subtypes. Thirdly, this study was unable to determine the effect of individual psychotropic medications on haemodynamic response. Contrary to the present study, Takamiya *et al*.^53^ observed an association between oxy-haemoglobin at 5 channels across the frontotemporal region and daily defined dose of antidepressants. As antidepressants can improve depressive symptoms^54^ and improve cognitive function^55^, future fNIRS studies comparing antidepressants may provide insights into their mechanism of action. Lastly, majority of patients included in this study suffered from mild to moderate depression. Therefore, replication studies in severely depressed patients are needed to further support the fNIRS diagnostic paradigm. Despite these limitations, this is the first study to validate a single trial fNIRS VFT paradigm in the English language for MDD. Furthermore, the moderate sample size allows findings from this study to be generalised to other adult populations.

In conclusion, cortical oxy-haemoglobin changes measured by fNIRS provides a safe, non-invasive and direct measure of cerebral physiological function. Accordingly, diminished haemodynamic response in the frontal and temporal cortices of patients with MDD provides further evidence for the biological basis of this disorder. Threshold integral values determined in this study enable this fNIRS paradigm to be used as an objective and quantitative assessment of psychiatric patients who present with depressive symptoms.

## Materials and methods

### Participants

One hundred and five HCs and 105 age- and gender-matched patients with MDD participated in this study. All participants spoke English and were between 21–65 years old. Depressive symptoms and psychosocial functioning for each participant were evaluated on the day of participation using the 17-item Hamilton rating scale for depression (HAM-D)^56^ and global assessment of functioning (GAF)^57^, respectively.

HCs recruited from the community were assessed by a psychiatrist, and those included in this study were certified as being normal. HCs did not have a history of any psychiatric illnesses, including alcohol/substance abuse or addiction. HCs were excluded if they had conditions that could affect the central nervous system, including neurological illnesses such as epilepsy, traumatic brain injury, cerebrovascular diseases, respiratory diseases, hepatic diseases, kidney diseases, cancer or intellectual disability. Additionally, HCs who received psychotherapy in the past, had a HAM-D score of 8 or higher on the day of participation^32^, or reported drowsiness on the day of participation, were excluded.

Patients with MDD were recruited from the outpatient psychiatric clinic at the National University Hospital, Singapore. They were diagnosed by a psychiatrist, according to the DSM-5 criteria^58^. Patients with MDD were excluded if they had any neurological illnesses, traumatic brain injury, cerebrovascular diseases, respiratory diseases, hepatic diseases, kidney diseases, cancer, intellectual disability or alcohol/substance abuse or addiction. In addition, patients with MDD who received psychotherapy in the past, and those who reported drowsiness on the day of participation, were excluded.

Study details were fully explained to participants, and their written informed consent was obtained. This study was performed according to the Declaration of Helsinki, and the ethical principles in the Belmont Report. It was approved by the Domain Specific Review Board of the National Healthcare Group, Singapore (protocol number 2017/00509).

### Verbal fluency task

**Figure 3:**
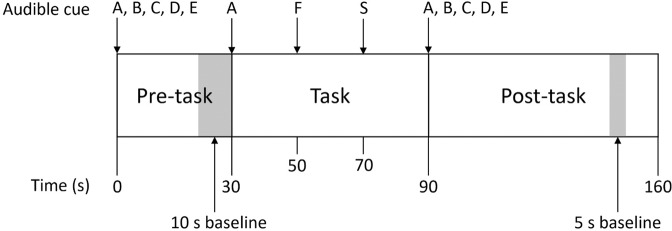
The verbal fluency task protocol.

Prior to the NIRS measurement recording, participants watched a demonstration video, in which they were asked to remain seated, avoid excessive body or head movements, and focus on a cross displayed during the VFT. The paradigm used in previous studies^18^ was modified for the English language (Fig. 3). It consisted of a 30 s pre-task period, 60 s task period, and a 70 s post-task period. During the pre- and post-task periods, participants were asked to say “A, B, C, D, E” aloud and repeatedly. During the task period, they were instructed to generate as many words as possible, beginning with A, F and S for 20 s per letter, similar to the conventional VFT^59^ and VFT protocols used in other fNIRS studies^60^. The total number of unique words, enunciated within the task period, was recorded as the task performance. Before the actual trial, participants were asked to practice the VFT for a shorter duration, and with the letters H, B and P. This ensured all participants understood the task and responded to the cues correctly during the actual trial.

### NIRS measurement

A 52-channel NIRS system (ETG-4000. Hitachi Medical Co., Tokyo, Japan) measured relative oxy-haemoglobin and deoxy-haemoglobin changes using 2 NIR light wavelengths (695 and 830 nm)^61^. Emitter and detector optodes were arranged 3 cm apart. The area between each emitter and detector pair is called a channel. Anatomically, channels correspond to cortical regions 2–3 cm beneath the skin and scalp surface^62^. Optodes were placed on the forehead and scalp, with the lowest optodes placed along the T4-Fpz-T3 line, defined by the 10/20 system. This arrangement allowed for haemoglobin changes in the bilateral prefrontal cortex, frontopolar cortex, and the anterior regions of the superior and middle temporal cortices to be measured. These approximate channel locations are based on the anatomical craniocerebral correction of the international 10/20 system. Real coordinates of each channel were acquired using a 3D digitiser (Patriot system, Polhemus, USA) for each subject. Coordinates were superimposed on a cerebral cortex atlas using the statistical parametric mapping for near-infrared spectroscopy (NIRS-SPM) toolbox^63–65^, and did not differ between controls and patients (Supplementary Table 1).

### NIRS signal analysis

**Figure 4:**
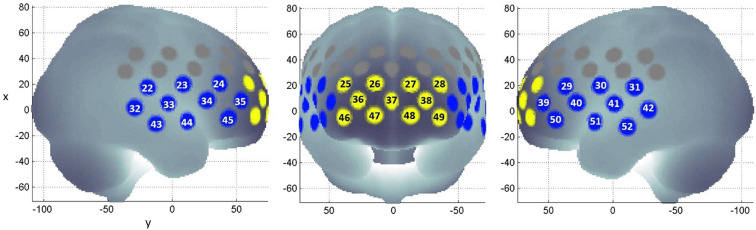
Channel positions within the frontal (yellow) and temporal (blue) regions of interest, plotted using NFRI functions toolbox^68,69^.

NIR signals were processed according to the method described by Takizawa *et al*.^18^. Oxy-haemoglobin, deoxy-haemoglobin and total haemoglobin were derived from optical densities using the modified Beer-Lambert law. Haemoglobin changes during the task period were normalised by linear fitting between a 10 s baseline at the end of the pre-task period, and a 5 s baseline 50 s into the post-task period (Fig. 3). A moving average factor of 5 was applied to remove short term motion artefacts. An algorithm identifying channels with body movement artefacts, or high and low frequency noise was applied. Artefact channels were not used in further analysis. Previous studies have also established the reliability of synthesising NIRS signals from a cluster of associated channels^19^. The first cluster of 11 channels is located approximately at the frontopolar and dorsolateral prefrontal cortex. The second cluster of 20 channels is located approximately at the left and right ventrolateral prefrontal cortex, superior temporal cortex, and middle temporal cortex. These clusters are referred to as ‘frontal region’ and ‘temporal region’ respectively (Fig. 4). Oxy-haemoglobin changes measured at the channels for each region are averaged for each subject. Participants with at least 6 available channels in either regions of interest were included. Oxy-haemoglobin changes at each region was quantified using 2 visual spatiotemporal indices, called the integral and centroid values. The integral value is the area under the oxy-haemoglobin curve during the task period, and it is a metric of haemodynamic response magnitude. The centroid value is the time corresponding to the centre of the area under the oxy-haemoglobin curve, and it is a metric of haemodynamic change over time. A small centroid value indicates an earlier haemodynamic response, while a large centroid value indicates a delayed haemodynamic response^19^.

### Statistical analysis

The effect of diagnostic group on continuous and categorical variables were determined using t-test and Pearson’s chi-square test, respectively. Continuous variables are age, years of education, number of words generated, HAM-D score, GAF score, integral values, centroid values and number of available channels. Categorical variables are gender, ethnicity, handedness and family psychiatric history. Integral and/or centroid values that were significantly different between MDD and HCs were used in further statistical analysis.

Variables associated with frontal and temporal region integral and/or centroid values within diagnostic groups were determined using Student’s t-test for dichotomous variables, Kruskal-Wallis test for categorical variables, and Pearson’s correlation for continuous variables. Dichotomous variables are gender, family psychiatric history, past admission to a psychiatric ward and pharmacotherapy. Categorical variables are ethnicity and handedness. Continuous variables are age, years of education, number of words generated, number of available channels in the corresponding region of interest, HAM-D score, GAF score, age at MDD onset, duration of MDD, and equivalent doses of antidepressants, anxiolytics and sedatives, and antipsychotics. Equivalent doses were calculated based on published mean dose ratios. Reference drugs for each class are fluoxetine, diazepam and chlorpromazine, respectively^66,67^. For patients receiving more than one drug in each class, the combined equivalent dose was calculated.

Subsequent stepwise linear regression was performed to verify variables associated with frontal and temporal region integral and/or centroid values. Independent variables included in the model for each diagnostic group are age, gender, years of education, HAM-D score, GAF score, number of words generated and number of available channels in the corresponding region of interest. Additional variables included in the regression analysis for patients with MDD are age at onset, duration of illness, past admission to a psychiatric ward and pharmacotherapy.

Lastly, receiver operating characteristic (ROC) analysis was performed to determine the accuracy of differentiating HCs and patients with MDD based on their frontal and temporal region integral and/or centroid value. All tests were two-tailed, with a significance level of *p* ≤ 0.05. Data are expressed as mean and standard deviation. The effect size (Cohen’s *d*) and the 95% confidence interval are reported wherever a statistically significant difference was observed between groups. Statistical analysis was done on SPSS Statistic 21.0 (IBM).

## Supplementary information

Supplementary Information.

## Data Availability

The data that support the findings of this study are available on request from the corresponding author. The data are not publicly available due to privacy or ethical restrictions.
