# Supplementary material for: Validating a functional near-infrared spectroscopy diagnostic paradigm for Major Depressive Disorder
Source: Sci Rep. 2020 Jun 16;10:9740. doi: 10.1038/s41598-020-66784-2 (PMC7298029; doi:10.1038/s41598-020-66784-2)
Supplement: Supplementary file 1 — Supplementary Information. [file 41598_2020_66784_MOESM1_ESM.pdf]

# Validating a functional near-infrared spectroscopy diagnostic paradigm for Major Depressive Disorder

Syeda F. Husain[1,2]; Rongjun Yu[3]; Tong-Boon Tang[4]; Wilson W. Tam[5]; Bach Tran[6,7,8]; Travis T. Quek[2]; Shi-Hui Hwang[2]; Cheryl W. Chang[9]; Cyrus S. Ho[2]; Roger C. Ho[1,2]*

[1] Institute for Health Innovation and Technology (iHealthtech), National University of Singapore, Singapore 117599, Singapore.

[2] Department of Psychological Medicine, Yong Loo Lin School of Medicine, National University of Singapore, Singapore 119228, Singapore.

[3] Department of Psychology, Faculty of Arts and Social Science, National University of Singapore, Singapore 119077, Singapore.

[4] Centre for Intelligent Signal and Imaging Research (CISIR), University Teknologi PETRONAS, 32610 Seri Iskandar, Perak Darul Ridzuan, Malaysia.

[5] Alice Lee Centre for Nursing Studies, Yong Loo Lin School of Medicine, National University of Singapore, Singapore 117597, Singapore.

[6] 1. Johns Hopkins Bloomberg School of Public Health, Johns Hopkins University, Baltimore, MD 21205, USA.

[7] Institute for Preventive Medicine and Public Health, Hanoi Medical University, Hanoi, Vietnam.

[8] Center of Excellence in Behavioral Medicine, Nguyen Tat Thanh University, Ho Chi Minh City, Vietnam.

[9] Department of Psychological Medicine, National University Health System, Singapore 119228, Singapore.

* **Correspondence:** Roger C. Ho, Institute for Health Innovation and Technology (iHealthtech), National University of Singapore, Singapore 117599, Singapore. (Email: pcmrhcm@nus.edu.sg)

**Supplementary table 1.** Channel coordinates of 46 healthy controls and 44 patients with major depressive disorder were compared using Student's t-test.

| Region of interest | Channel | Healthy control | | | Major depressive disorder | | | *p*-value | | |
|---|---|---|---|---|---|---|---|---|---|---|
| | | x | y | z | x | y | z | x | y | z |
| Frontal | 25 | 34.88 | 59.68 | 23.04 | 34.86 | 59.81 | 23.06 | 0.984 | 0.825 | 0.976 |
| | 26 | 14.14 | 68.98 | 23.54 | 14.27 | 68.83 | 23.93 | 0.821 | 0.695 | 0.624 |
| | 27 | -12.14 | 68.05 | 23.61 | -11.36 | 67.72 | 24.26 | 0.237 | 0.438 | 0.433 |
| | 28 | -34.28 | 57.76 | 23.59 | -33.24 | 57.73 | 24.75 | 0.199 | 0.969 | 0.151 |
| | 36 | 26.28 | 69.77 | 10.17 | 26.17 | 69.79 | 10.70 | 0.855 | 0.955 | 0.489 |
| | 37 | 0.97 | 70.59 | 10.14 | 1.72 | 70.28 | 10.80 | 0.492 | 0.401 | 0.377 |
| | 38 | -24.85 | 68.69 | 10.35 | -23.89 | 69.05 | 11.60 | 0.207 | 0.366 | 0.118 |
| | 46 | 38.30 | 64.52 | -3.92 | 38.39 | 64.39 | -3.50 | 0.896 | 0.802 | 0.594 |
| | 47 | 14.89 | 72.75 | -3.26 | 15.14 | 72.83 | -2.22 | 0.692 | 0.506 | 0.163 |
| | 48 | -13.38 | 72.53 | -3.17 | -12.73 | 72.71 | -2.08 | 0.314 | 0.339 | 0.138 |
| | 49 | -38.25 | 61.93 | -4.49 | -37.40 | 62.71 | -3.27 | 0.199 | 0.142 | 0.108 |
| Temporal | 22 | 68.99 | -10.74 | 28.33 | 69.00 | -10.90 | 27.23 | 0.980 | 0.900 | 0.341 |
| | 23 | 61.65 | 17.28 | 24.72 | 62.01 | 17.01 | 24.02 | 0.476 | 0.824 | 0.440 |
| | 24 | 50.96 | 42.04 | 23.78 | 51.25 | 41.86 | 23.33 | 0.563 | 0.837 | 0.585 |
| | 29 | -51.11 | 36.74 | 22.77 | -50.51 | 36.94 | 23.96 | 0.317 | 0.848 | 0.177 |
| | 30 | -61.74 | 10.78 | 22.30 | -61.31 | 10.98 | 23.67 | 0.396 | 0.877 | 0.151 |
| | 31 | -67.82 | -16.76 | 23.29 | -67.52 | -16.48 | 25.20 | 0.245 | 0.852 | 0.162 |
| | 32 | 71.64 | -26.50 | 13.60 | 71.69 | -26.62 | 12.44 | 0.825 | 0.931 | 0.439 |
| | 33 | 66.75 | 3.04 | 12.76 | 66.63 | 2.75 | 11.76 | 0.752 | 0.821 | 0.383 |
| | 34 | 58.81 | 32.33 | 10.97 | 58.86 | 32.07 | 10.30 | 0.896 | 0.793 | 0.460 |
| | 35 | 46.46 | 55.12 | 9.99 | 46.61 | 54.92 | 9.79 | 0.777 | 0.796 | 0.807 |
| | 39 | -46.93 | 50.38 | 8.79 | -46.16 | 51.09 | 10.39 | 0.188 | 0.394 | 0.068 |
| | 40 | -57.54 | 26.66 | 9.03 | -57.64 | 26.68 | 10.17 | 0.726 | 0.984 | 0.200 |
| | 41 | -65.46 | -3.18 | 7.49 | -65.22 | -2.66 | 9.45 | 0.512 | 0.696 | 0.160 |
| | 42 | -69.97 | -30.70 | 7.20 | -69.85 | -30.67 | 9.20 | 0.541 | 0.981 | 0.259 |
| | 43 | 71.24 | -12.91 | -3.93 | 71.18 | -13.11 | -4.68 | 0.848 | 0.867 | 0.581 |
| | 44 | 60.07 | 16.20 | -2.19 | 60.09 | 16.39 | -2.73 | 0.975 | 0.905 | 0.668 |
| | 45 | 53.74 | 44.92 | -3.80 | 53.78 | 44.56 | -3.92 | 0.926 | 0.700 | 0.877 |
| | 50 | -52.96 | 40.31 | -4.99 | -53.04 | 40.92 | -3.97 | 0.802 | 0.472 | 0.193 |
| | 51 | -59.89 | 9.47 | -7.25 | -59.60 | 10.09 | -5.43 | 0.633 | 0.656 | 0.213 |
| | 52 | -69.56 | -16.50 | -9.30 | -69.57 | -16.25 | -7.61 | 0.972 | 0.845 | 0.276 |

**Supplementary table 2.** Medication details.

| | n | Dose (mg/day) |
|---|---|---|
| Antidepressants | 66 | |
| *Selective serotonin reuptake inhibitors* | | |
| Escitalopram | 3 | 10 ± 0 |
| Fluoxetine | 10 | 22 ± 6.3 |
| Fluvoxamine | 22 | 72.7 ± 40 |
| Paroxetine | 6 | 20.8 ± 6.5 |
| Sertraline | 5 | 100 ± 50 |
| *Serotonin and norepinephrine reuptake inhibitors* | | |
| Duloxetine | 2 | 30 ± 0 |
| Venlafaxine | 2 | 150 ± 106.1 |
| *Noradrenergic and specific serotonergic antidepressant* | | |
| Mirtazapine | 14 | 25.7 ± 9.2 |

| | | |
|---|---|---|
| *Dopamine Norepinephrine Reuptake Inhibitor* | | |
| Bupropion | 7 | 150 ± 0 |
| *Tricyclic Antidepressants* | | |
| Amitriptyline | 1 | 25 |
| *Other antidepressants* | | |
| Agomelatine | 4 | 37.5 ± 14.4 |
| Vortioxetine | 4 | 8.7 ± 2.5 |
| *Combination antidepressants* | 13 | |
| Agomelatine & Bupropion | 2 | |
| Agomelatine & Sertraline | 1 | |
| Amitriptyline & Fluvoxamine | 1 | |
| Bupropion & Escitalopram | 1 | |
| Bupropion & Fluvoxamine | 1 | |
| Bupropion, Fluoxetine & Fluvoxamine | 1 | |
| Bupropion & Mirtazapine | 1 | |
| Duloxetine & Fluvoxamine | 1 | |
| Mirtazapine & Paroxetine | 2 | |
| Mirtazapine & Vortioxetine | 2 | |
| Antipsychotics | 16 | |
| Haloperidol | 1 | 1.5 |
| Olanzapine | 2 | 3.7 ± 1.8 |
| Quetiapine | 12 | 138.1 ± 97 |
| Risperidone | 1 | 2 |
| Anxiolytics, hypnotics and sedatives | 21 | |
| Alprazolam | 10 | 0.5 ± 0.4 |
| Clonazepam | 4 | 0.62 ± 0.2 |
| Diazepam | 1 | 5 |
| Lorazepam | 2 | 0.7 ± 0.3 |
| Zopiclone | 4 | 11.2 ± 4.3 |
| Zolpidem | 1 | 12.5 |
| *Combination Anxiolytics, hypnotics and sedatives* | | |
| Clonazepam & Zolpidem | 1 | |

**Supplementary table 3.** Determining variables associated with integral values for healthy controls.

| | Frontal region integral | | Temporal region integral | |
|---|---|---|---|---|
| | Test statistic | *p*-value | Test statistic | *p*-value |
| Student's t-test | | | | |
| Gender | 1.32 | 0.194 | 0.87 | 0.389 |
| Family psychiatric history | -0.25 | 0.805 | -0.58 | 0.566 |
| Kruskal-Wallis test | | | | |
| Ethnicity | 1.33 | 0.263 | 1.67 | 0.164 |
| Handedness | 0.52 | 0.598 | 1.73 | 0.184 |
| Pearson's correlation | | | | |
| Age (years) | -0.033 | 0.743 | -0.09 | 0.364 |
| Education (years) | 0.09 | 0.368 | 0.123 | 0.196 |

| | | | | |
|---|---|---|---|---|
| Number of words | -0.07 | 0.49 | -0.095 | 0.338 |
| Number of available channels | -0.16 | 0.11 | -0.22 | 0.024 |
| HAM-D score | -0.056 | 0.578 | -0.072 | 0.467 |
| GAF score | 0.15 | 0.127 | 0.167 | 0.091 |

**Supplementary table 4.** Determining variables associated with integral values for patients with major depressive disorder.

| | Frontal region integral | | Temporal region integral | |
|---|---|---|---|---|
| | Test statistic | *p*-value | Test statistic | *p*-value |
| Student's t-test | | | | |
| Gender | 1.2 | 0.234 | -0.037 | 0.971 |
| Family psychiatric history | 0.62 | 0.538 | 1.12 | 0.264 |
| Past admission | -0.81 | 0.42 | -1.18 | 0.241 |
| Pharmacotherapy | 0.16 | 0.871 | -0.36 | 0.724 |
| Kruskal-Wallis test | | | | |
| Ethnicity | 1.1 | 0.355 | 0.64 | 0.594 |
| Handedness | 0.025 | 0.975 | 0.65 | 0.527 |
| Pearson's correlation | | | | |
| Age (years) | -0.17 | 0.084 | -0.12 | 0.222 |
| Education (years) | 0.054 | 0.589 | 0.07 | 0.488 |
| Number of words | -0.078 | 0.438 | 0.12 | 0.223 |
| Number of available channels | -0.036 | 0.724 | -0.19 | 0.061 |
| HAM-D score | -0.03 | 0.766 | -0.019 | 0.848 |
| GAF score | 0.15 | 0.13 | 0.1 | 0.313 |
| Age at MDD onset (years) | -0.094 | 0.35 | -0.074 | 0.457 |
| Duration of MDD (years) | -0.18 | 0.073 | -0.11 | 0.263 |
| Fluoxetine equivalent dose (mg/day) | -0.2 | 0.109 | -0.087 | 0.493 |
| Diazepam equivalent dose (mg/day) | -0.38 | 0.107 | -0.33 | 0.141 |
| Chlorpromazine equivalent dose (mg/day) | -0.29 | 0.298 | -0.17 | 0.534 |
